# Ultimate Taipan with Symbolic Interpretation and Fluid Abstractions (Competition Contribution)

**DOI:** 10.1007/978-3-030-45237-7_32

**Published:** 2020-03-13

**Authors:** Daniel Dietsch, Matthias Heizmann, Alexander Nutz, Claus Schätzle, Frank Schüssele

**Affiliations:** 8 Johannes Kepler University, Linz, Austria; 9 University of Birmingham, Birmingham, UK; University of Freiburg, Freiburg im Breisgau, Germany

## Abstract

Ultimate Taipan is a software model checker that combines trace abstraction with abstract interpretation on path programs. In this year’s version, we replaced our abstract interpretation engine and now use a combination of multiple abstraction functions, fixpoint computation, algebraic program analysis, and SMT solving. Our new approach will allow us to integrate new techniques more easily.

## 1 Verification Approach

Ultimate Taipan is a software model checker which combines trace abstraction [8] and abstract interpretation [5]. The algorithm of Taipan follows the trace abstraction verification scheme for reachability where it constructs an abstraction of the program as a nested word automaton (NWA). This NWA has initially the same graph structure as the program's interprocedural control flow graph (ICFG), its states are program locations, its transitions are labeled with program locations, and states corresponding to error locations are accepting. Hence, the automaton recognizes a language where the symbols are statements and the words are sequences of statements (which we call traces) that lead to an error location. If the language of the abstraction automaton is empty, no error location can be reached and the program is safe. If there is a trace in the language, the algorithm needs to determine if it is a *feasible* trace, i.e., a trace that corresponds to an actual program execution, or not. Feasible traces constitute an actual counterexample and if one is found the algorithm terminates. If an infeasible trace is found, Taipan's algorithm differs from trace abstraction and does not only analyze the actual trace, but rather constructs a path program[1] from this trace. It then tries to synthesize inductive invariants for the whole path program [7]. From these invariants, a new automaton is constructed which language only recognizes infeasible traces. The new abstraction is then constructed as the difference of the automaton that only recognizes infeasible traces and the old abstraction automaton. If the error location's invariant of the path program is not $false$, the computed invariants are too weak to prove infeasibility, and Taipan falls back to using interpolating SMT solvers to compute new invariants that are strong enough to discharge the trace.

Daniel Dietsch — Jury Member

[1] A path program is a projection of the program to the trace.

Taipan's old algorithm used abstract interpretation to analyze path programs. In this year's iteration, we use a new approach, which is motivated by two drawbacks of our old algorithm. Firstly, extending an abstract interpretation engine with new abstract domains is labor-intensive and error-prone. Each abstract domain has an abstract post operator describing the effect program statements have on abstract states. For each abstract domain and each type of program statement the abstract post operator has to be defined and implemented, and re-use between domains is complicated. Furthermore, each abstract domain needs their own representation of an abstract state, s.t. exchanging information between multiple domains requires explicit conversions. Secondly, Abstract interpretation always abstracts. Because each abstract domain has its own abstract state representation, it is usually not possible to implement a precise post operator. Hence, every application of post is an abstraction, which leads to unnecessary loss of precision.

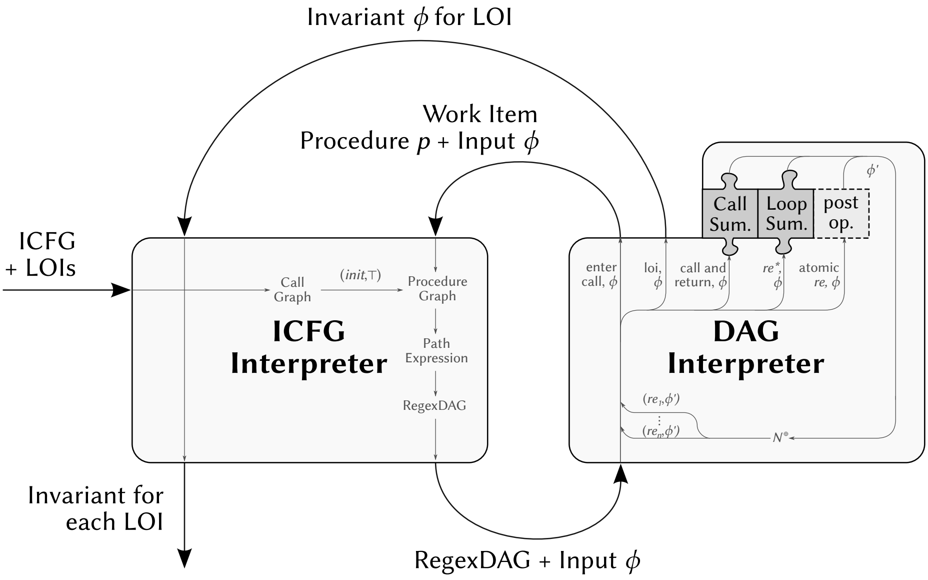

**Fig. 1:** Overview of the symbolic interpretation engine.

Our new approach is inspired by *Algebraic Program Analysis* [9, 4] and the renewed interest in this technique (e.g. [6]), and *Logical Interpretation* [10]. We use the modularity of algebraic program analysis to combine different techniques in an unifying framework and the idea of a shared representation of abstract program states as SMT formulas over which abstraction operators can compute fixpoints from logical interpretation.

An overview of our approach is depicted in Figure 1. The approach consists of two major components, the ICFG interpreter and the DAG interpreter.

The ICFG interpreter component generates for a (partial) interprocedural control flow graph (ICFG) and a subset of its program locations (locations of interest, LOI) a set of path expressions represented as RegexDAGs. A RegexDAG is a directed acyclic graph with vertices that are labeled with regular expressions over the program's statements without calls and returns but with summary and enter statements. Each RegexDAG has exactly one sink node that represents a location of interest. We use summary statements when we call to and return from a procedure on a path to a LOI, and enter statements when we do not return until we reach the LOI.

The DAG interpreter component then analyses a RegexDAG in topological order by applying different operators (Call Sum., Loop Sum., post op.) to the different vertex labels. All operators take a program state expressed as SMT formula $\phi$ and a regular expression over program statements (i.e., a vertex label) and produce a new (possibly abstracted) program state that captures all the effects. If a vertex has multiple incoming edges, the different input states are simply joined with a logical disjunction ($\vee$). Some of these operators depend again on the ICFG interpreter to compute their result. The most basic operator is the post operator (post op.), which computes strongest post for star-free regular expressions and optionally applies an abstraction function to the result. The choice of abstraction function and if to apply them is governed by different heuristics that can be changed. We call these heuristics *fluids*. The other operators are the call summarization (Call Sum.) and loop summarization (Loop Sum.) operators. The call summarization operator computes a summary for a procedure call, either with or without considering the context. The loop summarization operator computes a summary for the Kleene-star operator of regular expressions. Our current implementation does this by computing a fixpoint and resolving nested loops by recursively inserting summaries. The different operators (post, call summarization, loop summarization) are completely modular and can be considered black-boxes for the interplay between the two main components. When the DAG interpreter reaches the sink vertex of the RegexDAG, it returns the disjunction of this sink's input program states as invariant for this LOI.

## 2 Strengths and Weaknesses

Our new approach is easy to extend with new abstraction functions, fluids, and loop acceleration techniques. Compared to the previous approach we also gain much more precision by, e.g., having a reduced product between different kinds of abstraction without writing a transformation function – we can just use the logical disjunction. Using SMT formulas as representation of program states also allows us to reuse many of Ultimate's existing tools that deal with SMT, in particular simplification, quantifier elimination,, rewriting, and debugging.

Nevertheless, our current implementation is not as effective as the old one, because we did not finish porting the various abstract domains. We currently only support a basic interval abstraction and an explicit value abstraction, which severely limits the efficiency of our approach. We are also missing more intricate loop acceleration implementations, optimized fluid configurations, and our implementation does not yet support recursion.

## 3 Architecture, Setup, Configuration, and Project

ULTIMATE TAIPAN is a part of the open-soure program analysis framework ULTIMATE[2,3], written in Java and licensed under LGPLv3[4]. We used TAIPAN version 0.1.25-f470102c in our competition submission, which is available as a `.zip` archive from multiple sources[5,6,7]. Our submission requires Java 1.8 and Python 3.x. The submission contains an executable version of TAIPAN for Linux platforms, the binaries of the required SMT solvers Z3[8], CVC4[9], and MATHSAT[10], as well as a Python script, `Ultimate.py`, which maps the SV-COMP interface to ULTIMATE's command line interface. TAIPAN is invoked with

```
./Ultimate.py --spec prop.prp --file input.c --architecture
                  32bit|64bit --full-output
```

where `prop.prp` is the SV-COMP property file, `input.c` is the input C file, `32bit` or `64bit` is the architecture, and `--full-output` enables verbose output to `stdout`. The output of TAIPAN is written to the file `Ultimate.log`. A violation [3] or correctness [2] witness may be written to the file `witness.graphml`. The benchmarking tool BENCHEXEC [1] supports TAIPAN through the tool-info module `ultimatetaipan.py`[11]. TAIPAN participates in all categories, as declared in its SV-COMP benchmark definition file `utaipan.xml`[12].
